# Zn-Induced Synthesis of Porous Fe-N,S-C Electrocatalyst with Iron-Based Active Sites Containing Sulfides, Oxides and Nitrides for Efficient Oxygen Reduction and Zinc-Air Batteries

**DOI:** 10.3390/molecules28155885

**Published:** 2023-08-04

**Authors:** Haiyan Zhao, Li Chen, Nan Ni, Yang Lv, Hezhen Wang, Jia Zhang, Zhiwen Li, Yu Liu, Yubo Geng, Yan Xie, Li Wang

**Affiliations:** 1 Liaoning Key Laboratory of Plasma Technology, School of Physics and Materials Engineering, Dalian Minzu University, 18 Liaohe West Road, Dalian 116600, China; zhaohaiyan@dlnu.edu.cn (H.Z.); nanni_1100@126.com (N.N.); tsblike@126.com (Y.G.); 2 Shanghai Key Laboratory of Molecular & Process Engineering, School of Chemistry and Molecular Engineering, East China Normal University, 3663 North Zhongshan Road, Shanghai 200062, China; chenli@chem.ecnu.edu.cn; 3 State Key Laboratory of Fine Chemicals, School of Chemical Engineering, Dalian University of Technology, 2 Linggong Road, Dalian 116024, China; yanglv@dlut.edu.cn; 4 Dalian Institute of Chemical Physics, Chinese Academy of Sciences, 457 Zhongshan Road, Dalian 116023, China; hzwang@dicp.ac.cn (H.W.); zhangjia610@dicp.ac.cn (J.Z.); yuliu@dicp.ac.cn (Y.L.); 5 College of Chemistry and Materials Science, Inner Mongolia Minzu University, No. 536 West Huolinhe Road, Tongliao 028000, China

**Keywords:** FeNSC, hemin, oxygen reduction reaction, Zn–air battery

## Abstract

There is an urgent need to design and synthesize non-noble metal electrocatalysts (NNMEs) for the replacement of platinum-based electrocatalysts to enhance the sluggish oxygen reduction reaction (ORR) for Zn–air batteries and fuel cells. Herein, Fe-N,S-C materials were fabricated through two steps: first, reprecipitating hemin by adjusting the pH and, then, decorating it with melamine and cysteine in the presence of Zn^2+^. The resulting Fe-N,S-C-950 (Zn) was prepared after pyrolysis at 950 °C. Using this method, abundant iron-based active species with good dispersion were obtained. The fabrication of more micropores in Fe-N,S-C-950 (Zn) plays a positive role in the improvement of ORR activity. On comparison, Fe-N,S-C-950 (Zn) outperforms Fe-N,S-C-950 and Fe-N-C-950 (Zn) with respect to the ORR due to its larger specific surface area, porous structure, multiple iron-based active sites and N- and S-doped C. Fe-N,S-C-950 (Zn) achieves outstanding ORR performances, including a half-wave potential (E_1/2_) of 0.844 V and 0.715 V versus a reversible hydrogen electrode (RHE) in 0.1 M KOH and 0.1 M HClO_4_ solution, respectively. In addition, Fe-N,S-C-950 (Zn) shows an outstanding Zn–air battery performance with an open-circuit voltage (OCV) of 1.450 V and a peak power density of 121.9 mW cm^−2^, which is higher than that of 20 wt% Pt/C. As a result, the as-prepared electrocatalyst in this work shows the development of the Zn-assisted strategy combined with the assembly of porphyrins as NNMEs for the enhancement of the ORR in both alkaline and acidic solutions.

## 1. Introduction

Currently, noble metal electrocatalysts (NMEs) are being efficiently utilized for the electrochemical reduction of oxygen, namely the oxygen reduction reaction (ORR), in fuel cells (FCs) and Zn-air batteries (ZABs) [1,2]. However, the multiple disadvantages of Pt, such as high cost, scarcity and susceptibility to CO poisoning severely impede the development of commercial applications of FCs and ZABs. Therefore, it is of great significance to explore cost-effective, high-active ORR electrocatalysts, especially using earth-abundant materials, like non-noble metal electrocatalysts (NNMEs), as substitutes for NMEs [3,4]. Recently, due to tremendous constructive achievements, materials comprising the form Me-N-C (Me = transition metal, like Fe, Co, Mn, Ni and Zn) have been demonstrated as attractive NNMEs that favor the ORR [5,6,7]. Among them, Fe and/or Co are regarded as the most active ion centers for ORR electrocatalysts [8]. In recent decades, the literature has confirmed that single-atom catalysts (SACs) full of metal nitride (MeN_x_) sites exhibit superior catalytic efficiency relative to their cluster and nanoparticle (NP) counterparts in primary structures [9]. Nonetheless, to increase the content of the transition metal, in most cases, various transition metal-based active sites, like oxides, carbides and nitrides in an electrocatalyst, play a crucial role in the synergistic effects for the enhancement of ORR activities [10,11].

In 2009, Dai showed the first N-doped carbon (N-C) materials with well-identified N-based active sites, displaying a new catalytic mechanism of doping-induced charge transfer [12]. Tremendous efforts have been focused on this worldwide interest in N-C materials [13,14,15]. Based on recent studies, it was revealed that MeN_x_ with a non-uniform charge distribution can better adsorb/desorb oxygen intermediates [16,17]. Thus, to regulate the electronic states of MeN_x_, it is important to break the balance charge distribution. And the effective way is to adjust the charge distribution of MeN_x_ by introducing a second doping heteroatom, especially S, which is significant for creating defects in the carbon matrixes and increasing the metal sulfides (namely MeS_x_) in NNMEs [18,19]. Meanwhile, the introduction of S further modulates the electron cloud distribution around Me-N sites, where thiophene-S can enhance the adsorption of OH^−^ to OOH^−^ to yield higher reaction kinetics [20,21]. Thanks to the S doping within the N-C matrixes, N and S co-doped carbon (N,S-C) materials have been demonstrated to show comparable or even better catalytic performances than commercial Pt/C in alkaline media [22,23]. Additionally, as our previous work mentioned, Fe-N,S-C electrocatalysts indicated that the supplemental FeS_x_ indeed boosted the ORR performances relative to Fe-N-C [24,25]. In particular, Fe-N,S-C electrocatalysts with a higher content of Fe were investigated in the design and synthesis of promising NNMEs to boost ORR activities [26]. Commonly, NNMEs easily display outstanding ORR performances in alkaline solutions, but they have poor ORR activities in acidic solutions due to the different reaction mechanisms and different active sites in alkaline and acidic solutions. Therefore, it is crucial to investigate effective NNMEs with excellent ORR performances in both alkaline and acidic solutions.

Following Xie’s work in 2014 [27], it was found that Fe-based NPs obtained by the self-assembly of hemin became smaller with good dispersion compared with those obtained by the direct pyrolysis of pure hemin. Therefore, to pursue novel approaches to Fe-N,S-C electrocatalysts, we first assembled hemin on carbon. Then, cysteine and melamine, as N,S- and N-containing carbon resources, were used to modify the carbon-supported hemin in the presence of ZnCl_2_. Of note is that, in addition to the use of the Zn element, more micropores can also be created among hierarchical porous carbon structures by carbonization at 950 °C. As a result, the self-assembly of hemin is beneficial for obtaining a high density of Fe-based active sites with well-dispersed NPs within or on the N,S-C matrixes. In stark contrast, Fe-N,S-C-950 (Zn) shows excellent ORR performances in terms of an onset potential (E_onset_) of 0.921 V (vs. RHE) and a half-wave potential (E_1/2_) of 0.844 V (vs. RHE), which is superior to those of Fe-N,S-C-950 (E_onset_ = 0.888 V vs. RHE; E_1/2_ = 0.785 V vs. RHE) and commercial Pt/C (E_onset_ = 0.909 V vs. RHE; E_1/2_ = 0.837 V vs. RHE). Furthermore, the E_onset_ and E_1/2_ of Fe-N,S-C-950 (Zn) is about 0.818 V and 0.715 V (vs. RHE), indicating a good ORR performance in acidic solutions. More importantly, Fe-N,S-C-950 (Zn) still shows excellent stability after 5000 cyclic voltammetry (CV) cycles in an alkaline solution and 2500 cycles in an acidic solution. Such outstanding ORR performances are due to the multiple active sites and hierarchical porous structure of Fe-N,S-C-950 (Zn). The sacrificial template of Zn, the higher density of iron nitrides and the larger surface area of Fe-N,S-C-950 (Zn) further enhance the ZAB performance with a maximum power density of 121.9 mW cm^−2^, which is higher than that of 20 wt% Pt/C in 6 M KOH solution. This work provides an interesting strategy for the synthesis of N- and S-co-doped carbon materials, which can be used as a reference for the development of NNMEs for ZABs.

## 2. Results and Discussion

**Scheme 1:**
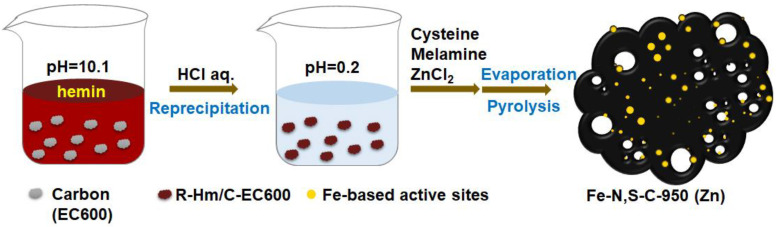
Schematic illustration for the preparation of Fe-N,S-C-950 (Zn).

As shown in Scheme 1, Fe-N,S-C-950 (Zn) was synthesized through two steps. First, hemin was self-assembled to reprecipitate on the EC600 commercial carbon (R-Hm/C-EC600) by adjusting the pH of the solution from 10.1 to 0.2. As a kind of low-cost and abundant macrocyclic compound, hemin has unique properties for the efficient preparation of ORR electrocatalysts [28]. It was found that the assembled hemin could further improve the dispersibility of NPs [27]. Then, N-/N,S-containing ligands, namely melamine and cysteine, were further used to be modified on R-Hm/C-EC600 in the presence of ZnCl_2_. The synergistic effect of melamine and cysteine could modulate the N-doped species within carbon matrixes, in which the Fe-N was known as one of the most effective actives for ORR. Additionally, the rich nitrogen-containing functional groups in melamine are regarded as an ideal N-containing precursor for NNMEs. And cysteine is a dual-doped ligand with both N and S elements for NNMEs. Notably, Zn was also applied for more accommodations for active sites among the carbon structure. In addition, the abundant N-containing ligand of melamine and the N,S-containing ligand of cysteine could contribute to anchor metal ions causing a high content of nitrides, sulfides, and N,S-C in the electrocatalysts [29]. Subsequently, the obtained samples were carbonized at 500 and 950 °C. In particular, Zn salts as pore-forming additives could be evaporated at 950 °C, making obvious defects among carbon matrixes [30]. Meanwhile, the resulting electrocatalysts of Fe-N,S-C-950 and Fe-N-C-950 (Zn) were synthesized under the same condition without the addition of ZnCl_2_ or cysteine.

**Figure 1:**
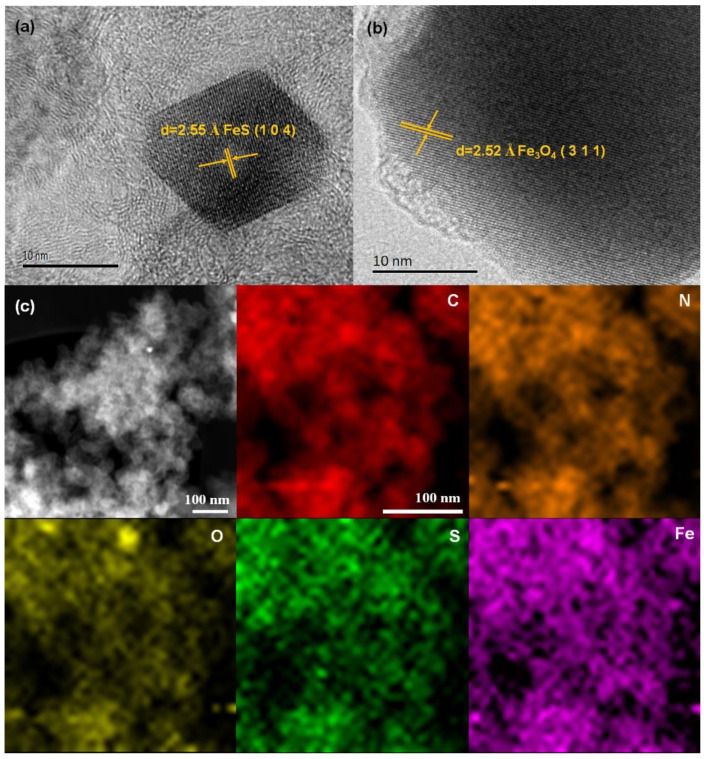
(**a**,**b**) the HR-TEM images, (**c**) HAADF-STEM image and its corresponding element mapping of C, N, O, S and Fe of Fe-N,S-C-950 (Zn).

As shown in Figure S1, the transmission electron microscopy (TEM) image of Fe-N,S-C-950 (Zn) indicates that NPs are well-dispersed on the C-EC600. The high-resolution TEM (HR-TEM) images clearly confirm that an interlayer spacing of 2.55 Å lattice is indexed to the (104) plane of FeS (Figure 1a). An interlayer spacing of 2.52 Å lattice is indexed to the (311) plane of Fe_3_O_4_ (Figure 1b). Energy-dispersive X-ray spectrometer (EDX) mapping of the representative region in the high-angle annular dark-field scanning transmission electron microscopy (HAADF-STEM, Figure 1c) of Fe-N,S-C-950 (Zn) confirms that N, O, S and Fe distribute uniformly throughout the entire carbon structures. It verifies that N and S are co-doping within carbon matrixes, which can effectively improve the conductivity of the carbon matrixes [31]. Moreover, the uniform signal of Fe element throughout the N,S-C substrates indicates the high density of Fe-based active sites with the Fe mass loading of 4.2 wt% that is assessed by the inductively coupled plasma optical emission spectrometer (ICP-OES).

**Figure 2:**
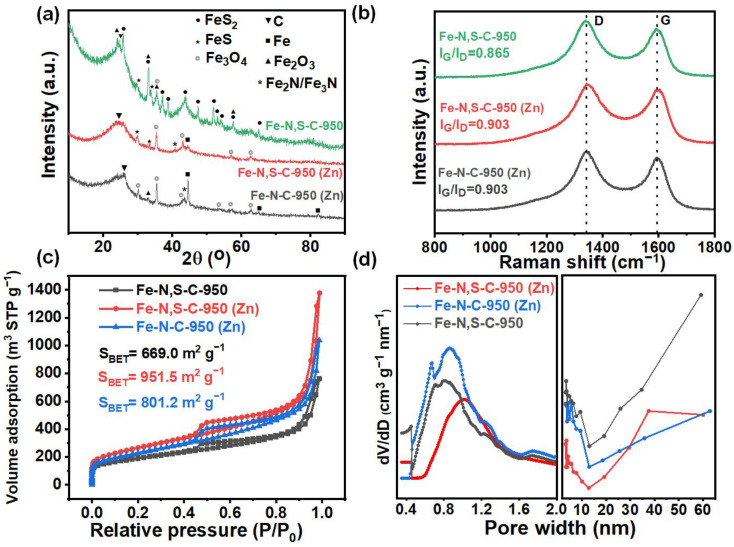
(**a**) XRD patterns, (**b**) Raman spectra, (**c**) N_2_ adsorption–desorption isotherms and (**d**) pore size distribution of Fe-N,S-C-950 (Zn), Fe-N,S-C-950 and Fe-N-C-950 (Zn).

Further investigation of the structure of electrocatalysts was detected by the powder X-ray diffraction (XRD) in Figure 2a. For Fe-N,S-C-950 (Zn), the wide diffraction peaks around 24.9° are assigned to C (PDF #74-2329), [32] confirming an obvious graphitic carbon after being treated at 950 °C. Those characteristic peaks at 30.1, 35.5, 43.1, 57.0 and 62.6° were identified as (220), (311), (400), (511) and (440) of Fe_3_O_4_ (PDF #89-0691) [33]. In addition, the obvious peaks around 29.8, 33.5 and 43.2° are characteristic of FeS (PDF #76-0961), [34] corresponding to (110), (112) and (114). The appearance of FeS and Fe_3_O_4_ detected by XRD is in accordance with the TEM results in Figure 1. Small peaks at 40.7 and 42.9° are the characteristic peaks of Fe_2_N (PDF #73-2102) [35]. A sharp peak around 44.6° belongs to Fe (PDF #85-1410) [36]. In contrast, the obvious peaks of FeS_2_ are observed in Fe-N,S-C-950. It can be seen that FeS_2_ can be further transferred to FeS in some cases. In this work, it may be attributed to the use of Zn. For Fe-N-C-950 (Zn), a characteristic peak around 26.3° can be assigned as (111) of C (PDF #75-0444) [37]. Multiple species like iron oxides, iron nitrides and Fe are also observed in Fe-N-C-950 (Zn). No more sulfides are detected in Fe-N-C-950 (Zn) without the addition of cysteine. Such obtained iron-based species, including oxides, nitrides and sulfides are commonly used as active sites in NNMEs for ORR and ZABs.

As shown in Figure 2b, there are two distinct peaks of electrocatalysts in Raman spectra. One peak around 1340 cm^−1^ (D-band) is disorder induced for the structural defects on the graphitic plane. The other peak around 1590 cm^−1^ (G-band) presents the graphitic plane for the E_2g_ vibrational mode in the sp^2^ banded graphitic carbon [38]. Noticeably, the Zn-induced method for the fabrication of Fe-N,S-C-950 (Zn) and Fe-N-C-950 (Zn) is beneficial for the graphite. As calculated, the I_G_/I_D_ ratio of Fe-N,S-C-950 (0.865) is lower than those of Zn-inducted electrocatalysts of Fe-N,S-C-950 (Zn) and Fe-N-C-950 (Zn), both of which are 0.903 with the similar amorphous carbon structure.

The specific surface areas and porosities of electrocatalysts were investigated by N_2_ adsorption–desorption isotherms (Figure 2c,d and Table S1). Obviously, type-IV isotherms with a more pronounced hysteretic loop of electrocatalysts indicate the co-existence of macro/meso/micro pores in the structures of Fe-N,S-C-950 (Zn) and Fe-N-C-950 (Zn). As shown in Table S1, Fe-N,S-C-950 (Zn) shows the largest Brunauer–Emmett–Teller (BET) surface area (S_BET_ = 951.5 m^2^ g^−1^) than Fe-N,S-C-950 (S_BET_ = 669.0 m^2^ g^−1^) and Fe-N-C-950 (Zn) (S_BET_ = 801.2 m^2^ g^−1^). In particular, the t-plot micropore area of Fe-N,S-C-950 (Zn) (S_Micro, BET_ = 172.8 m^2^ g^−1^) increases by using Zn as a micropore-introducing precursor. The microporous structure can provide numerous active sites for the enhancement of ORR performances [2]. The obtained porous structures can also be verified in the HR-TEM images of Fe-N,S-C-950 (Zn) in Figure S2. The micropore of Fe-N,S-C-950 (Zn) located around 1.02 nm is larger than those of Fe-N,S-C-950 and Fe-N-C-950 (Zn). The mesopore of Fe-N,S-C-950 (Zn) concentrates on 3.69 and 37.58 nm may enhance the mass/electron transport during the ORR process [39,40].

**Figure 3:**
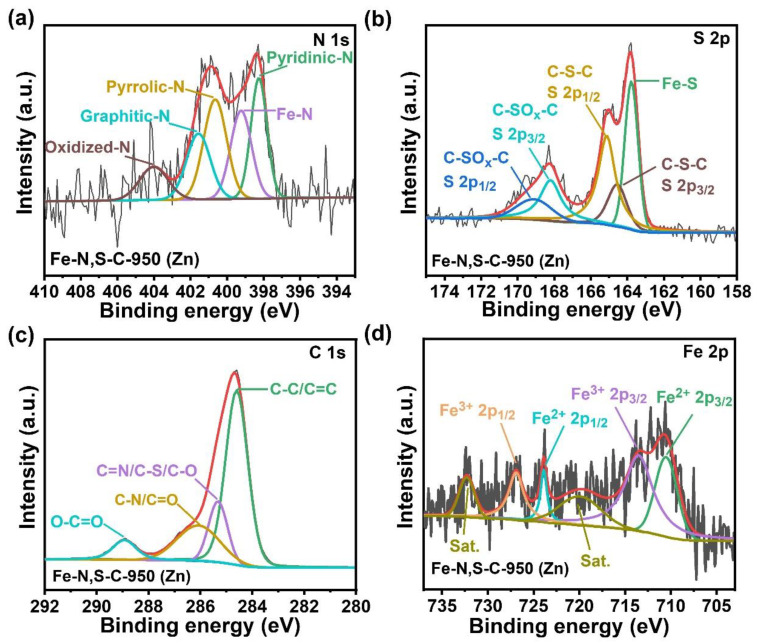
High-resolution XP spectra of (**a**) N 1s, (**b**) S 2p, (**c**) C 1s and (**d**) Fe 2p of Fe-N,S-C-950 (Zn).

The X-ray photoelectron (XP) spectroscopy confirms the elements and chemical states of electrocatalysts in Figure 3a,d, Figure S3, Figure S4 and Table S2. The high-resolution N 1s of as-prepared electrocatalysts can be deconvoluted into five peaks. As shown in Figure 3a, Figure S3 and Figure S4, the N 1s XP spectrum of Fe-N,S-C-950 (Zn) is divided as 398.3, 399.2, 400.7, 401.6 and 404.0 eV, corresponding to pyridinic N (23.0%), Fe-N (21.3%), pyrrolic-N (26.4%), graphitic-N (18.4%) and oxidized N (10.9%) [41]. Based on the data from Figures S3 and S4, the N relative content to C of Fe-N,S-C-950 (Zn) is determined to be 0.91%. Obviously, the higher content of graphitic-N in Fe-N,S-C-950 results in larger limiting current density (J_L_), while the higher content of Fe-N and pyrrolic-N in Fe-N,S-C-950 (Zn) is profound to enhance the ORR performances in an alkaline solution. This outcome is also inferred that the appearance of Zn in Fe-N,S-C-950 (Zn) (Fe-N, 21.3%) and Fe-N-C-950 (Zn) (Fe-N, 17.1%) is beneficial for retaining the component of Fe-N as the active sites for the enhancement of ORR performances. In the S 2p XP spectrum (Figure 3b), the fitting peak at 163.8 eV is corresponding to metal sulfides. Two peaks at 164.6 and 165.1 eV are attributed to S 2p_3/2_ and 2p_1/2_ electrons for C-S-C. Two peaks at 168.2 and 169.1 eV are consistent with S 2p_3/2_ and 2p_1/2_ electrons for C-SO_x_-C, respectively. The C 1s XP spectrum (Figure 3c) is deconvoluted into four peaks at 284.6, 285.4, 286.3 and 288.9 eV, which are associated with the sp^2^-C with C-C/C=C, C=N/C-S/C-O, C-N/C=O and O-C=O bonds, respectively [42]. As reported, the interface properties of the electrode can be further improved through the enhanced wettability aroused by these oxygen-containing groups on the surface of carbon materials [43]. The high-resolution Fe 2p XP spectrum of Fe-N,S-C-950 (Zn) (Figure 3d) shows that the binding energy at 710.5 and 723.9 eV are associated with Fe 2p_3/2_ and Fe 2p_1/2_ for Fe^2+^. Two peaks at 713.5 and 726.9 eV are associated with Fe 2p_3/2_ and Fe 2p_1/2_ for Fe^3+^ [44]. Two peaks at 719.9 and 732.3 eV are the satellite peaks [45]. It confirms the existence of Fe^3+^ and Fe^2+^, which is well aligned with the XRD patterns and TEM results.

**Figure 4:**
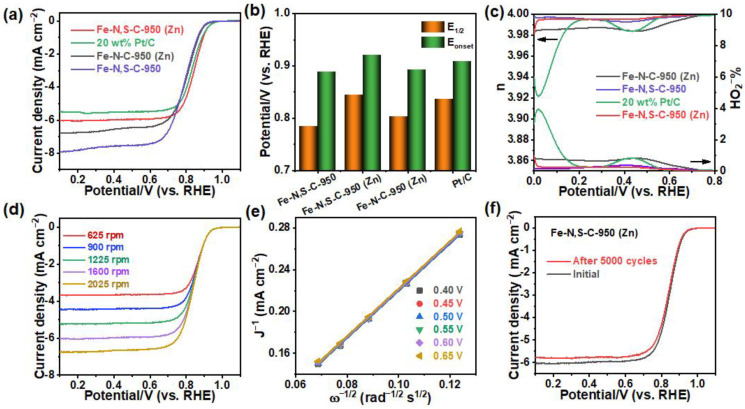
(**a**) The LSV curves; (**b**) the E_1/2_ and E_onset_; (**c**) n and HO_2_^−^% at 1.2 V (vs. RHE) of Fe-N-C-950 (Zn), Fe-N,S-C-950 (Zn), Fe-N,S-C-950 and 20 wt% Pt/C; (**d**) LSV curves of Fe-N,S-C-950 (Zn) with different rotation rates from 625 to 2025 rpm with a scanning rate of 5 mV s^−1^; (**e**) K-L plots based on the data from (**d**,**f**) LSV curves of Fe-N,S-C-950 (Zn) before and after 5000 CV curves at 1600 rpm in 0.1 M KOH solutions.

**Figure 5:**
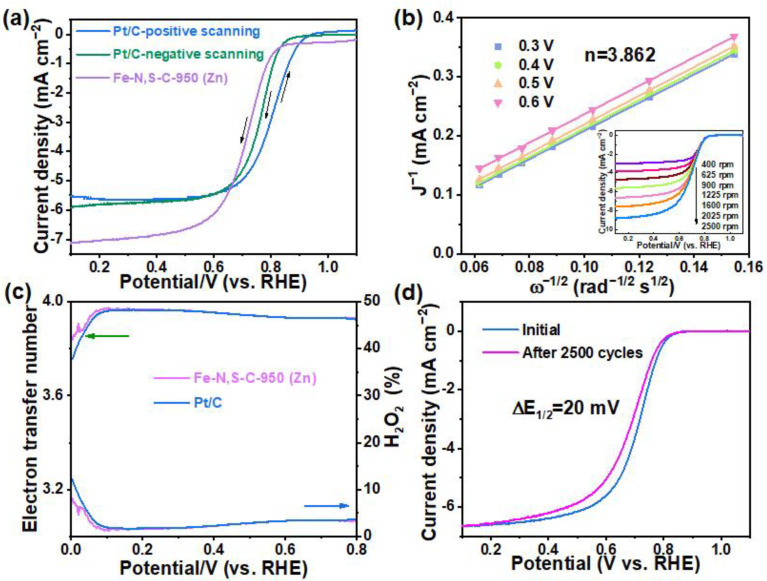
(**a**) The LSV curves of Fe-N,S-C-950 (Zn) and 20 wt% Pt/C with positive and negative scanning at 1600 rpm in 0.1 M HClO_4_ solutions. (**b**) The K-L plots at certain potentials from 0.3 to 0.6 V (vs. RHE) derived from LSV curves from 400 to 2500 rpm in the inset. (**c**) Electron transfer number and H_2_O_2_% of Fe-N,S-C-950 (Zn) and 20 wt% Pt/C. (**d**) The LSV curves of Fe-N,S-C-950 (Zn) before and after 2500 cycles at 1600 rpm in acidic solutions.

Here, we used the rotating disk electrode (RDE) technique to evaluate the ORR activities in the O_2_-saturated 0.1 M KOH solution. First, the cyclic voltammetry (CV) curves of Fe-N,S-C-950 (Zn) were conducted in the O_2_ and N_2_-saturated electrolytes, respectively (Figure S5). An oxygen redox peak was distinctly observed in the O_2_-saturated 0.1 M KOH solution. Then, we evaluated the electrocatalytic ORR performances of the resulting electrocatalysts and commercial Pt/C by the linear sweep voltammetry (LSV) in Figure 4a. In Figure 4b, Fe-N,S-C-950 shows an E_1/2_ of 0.785 V (vs. RHE) and an E_onset_ of 0.888 V (vs. RHE). After modified with Zn in Fe-N,S-C-950 (Zn), the ORR performances are enhanced with an E_1/2_ of 0.844 V (vs. RHE) and an E_onset_ of 0.921 V (vs. RHE) that is higher than 20 wt Pt/C (E_1/2_ = 0.837 V and E_onset_ = 0.909 V vs. RHE). Additionally, Fe-N,S-C-950 (Zn) demonstrates a J_L_ of −6.03 mA cm^−2^ at 0.1 V (vs. RHE). Fe-N-C-950 (Zn) without S doping displays a negative ORR performance with an E_1/2_ of 0.803 V (vs. RHE) and an E_onset_ of 0.893 V (vs. RHE), indicating that the S addition in electrocatalysts indeed boosts the ORR performances, probably due to the prominent contribution of FeS_x_. On one hand, the addition of S with the electronegativity of 2.58 around FeN_x_ results in an uneven charge distribution of FeN_x_ that is beneficial for the ORR process [31]. On the other hand, the porous structure of electrocatalysts is developed in the presence of Zn. The central atomic charge distribution of FeN_x_ improves because of the strong electronegativity of S. In the comparison with some reported work in Table S3, the as-prepared electrocatalyst in this work also shows comparable ORR performances in alkaline solutions. Moreover, the rotating ring-disk electrode (RRDE) was used to detect the average electron transfer number (n) and HO_2_^−^ yield of electrocatalysts in alkaline solutions. As a result, Fe-N,S-C-950 (Zn) is calculated to be around 3.996 (n = 3.989, Fe-N-C-950 (Zn); n = 3.996, Fe-N,S-C-950) from 0 to 0.8 V (vs. RHE) in Figure 3c. The HO_2_^−^ yield of Fe-N,S-C-950 (Zn) is lower than 0.26%, confirming a high selectivity with a four-electron pathway for the ORR process. The J_L_ of Fe-N,S-C-950 (Zn) increases from 625 to 2025 rpm in the O_2_-saturated 0.1 M KOH solution. Based on the data from Figure 4d, the Koutecky–Levich (K–L) plots were determined in Figure 4e, in which n of Fe-N,S-C-950 (Zn) is around 3.902. It is similar to the n value (3.996) derived from the RRDE. The stability of Fe-N,S-C-950 (Zn) was also measured in the O_2_-saturated 0.1 M KOH solution. The LSV curves were conducted and shown in Figure 4f before and after 5000 CV cycles at 1600 rpm in alkaline solutions. It implies that the E_1/2_ after 5000 CV cycles is about 0.849 mV. Only a slight loss of J_L_ happens after the continuous 5000 CV cycles in the range of 0.6 to 1.1 V (vs. RHE), further indicating good stability of Fe-N,S-C-950 (Zn) that is suitable for FCs. Furthermore, we also measured it in acidic solutions, in which the E_onset_ and E_1/2_ of Fe-N,S-C-950 (Zn) is 0.813 V and 0.715 V (vs. RHE) at a negative scanning, comparable to that of Pt/C in Figure 5a. The K-L plots in Figure 5b are derived from various LSV curves from 400 to 2500 rpm in the inset of Figure 5b. The calculated n is about 3.862, which is very close to that of commercial Pt/C in Figure 5c. This result demonstrates that the H_2_O_2_ yield of Fe-N,S-C-950 (Zn) and Pt/C are nearly lower than 2.3% in 0.4 V (vs. RHE) in Figure 5c. More importantly, the durability of Fe-N,S-C-950 (Zn) (Figure 5d) was conducted and only 20 mV loss happened after 2500 CV cycles in 0.1 M HClO_4_ solutions. Above all, the resulting electrocatalyst in this work shows excellent ORR performances in both alkaline and acidic solutions.

**Figure 6:**
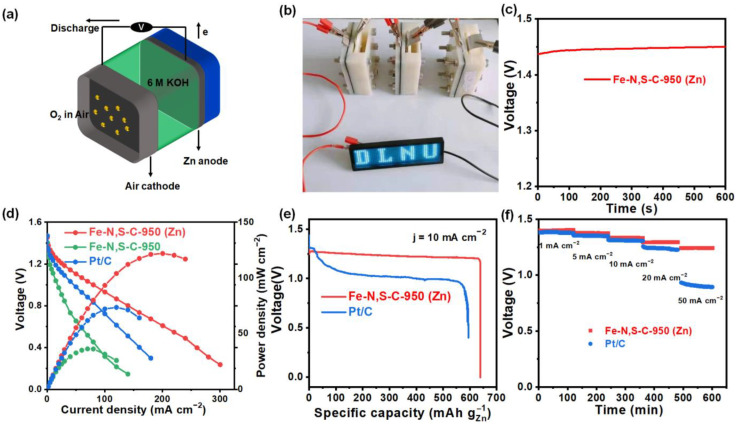
(**a**) A schematic illustration of the principle of a Zn-air battery (ZAB). (**b**) Photograph of blue LED screen powered by three Fe-N,S-C-950 (Zn)-based ZABs in series. (**c**) The open-circuit voltage (OCV) of ZAB for Fe-N,S-C-950 (Zn). (**d**) Polarization curves and the corresponding power density curves of the ZABs with the assembled electrocatalysts of Fe-N,S-C-950 (Zn), Fe-N,S-C-950 and 20 wt% Pt/C. (**e**) Specific capacity plots of Fe-N,S-C-950 (Zn) and Pt/C at 10 mA cm^−2^. (**f**) Discharge curves of Fe-N,S-C-950 (Zn) and Pt/C at different current densities of 1, 5, 10, 20 and 50 mA cm^−2^.

The ORR performance is about the discharge process ($\mathrm{Anode}\;\mathrm{reaction}:\;\mathrm{Zn}+4{\mathrm{OH}}^{-}-2e^{-}\overset{\mathrm{discharge}}{\to }\mathrm{Zn}{\left(\mathrm{OH}\right)}_{4}{}^{2-};\;\mathrm{Cathode}\;\mathrm{reaction}:{\;O}_{2}+2H_{2}O+4e^{-}\overset{\mathrm{discharge}}{\to }4{\mathrm{OH}}^{-}$; overall reaction: $\mathrm{Zn}+\frac{1}{2}O_{2}\overset{\mathrm{discharge}}{\to }\mathrm{ZnO}$) in the ZAB [46]. The schematic graph of ZAB is shown to evaluate the practical application of electrocatalysts for energy devices in Figure 6a. Three Zn-air cells were assembled in series with Fe-N,S-C-950 (Zn) as the cathode electrocatalyst, providing sufficient power for a light-emitting diode (LED) lighting up the blue “DLNU” characters (Figure 6b). The open-circuit voltage (OCV) of a Fe-N,S-C-950 (Zn) assembled battery is measured to be as high as 1.450 V in Figure 6c. Here, the commercial Pt/C electrocatalyst was also employed as a cathode electrocatalyst for ZABs for further comparison. The discharge polarization curves in Figure 6d disclose that the peak power density of Fe-N,S-C-950 (Zn) is around 121.9 mW cm^−2^, significantly higher than 36.2 mW cm^−2^ of Fe-N,S-C-950 and 73.5 mW cm^−2^ of 20 wt% Pt/C-based ZABs. In particular, the enhanced peak power density of Fe-N,S-C-950 (Zn) is comparable to most advanced NNMEs (Table S4). The formation of more triple-phase reaction interfaces and the fast mass transfer on the air cathode is further improved with a high ZAB performance, due to the high specific surface area, optimized pore structure and suitable doping of N and S in Fe-N,S-C-950 (Zn) [47]. As a result, the specific capacity of Fe-N,S-C-950 (Zn) is calculated to be 639 mA h g^−1^, which is higher than that of 20 wt% Pt/C (594 mA h g^−1^) at 10 mA cm^−2^ in Figure 6e. Remarkably, the ZABs catalyzed by Fe-N,S-C-950 (Zn) have a better rate performance in comparison with commercial Pt/C-based ZABs under the current density of 1 to 50 mA cm^−2^ (Figure 6f).

## 3. Materials and Methods

### 3.1. Materials

Hemin was purchased from Shanghai Aladdin Biochemical Technology Co., Ltd. (Shanghai, China). Melamine was purchased from Tianjin Kemiou Chemical Reagent Co., Ltd. (Tianjin, China). 2-Amino-3-mercaptopropanoic acid (cysteine) was obtained from Bide Pharmatech Ltd. (Shanghai, China). Zinc chloride was purchased from Damao Chemical Reagent Factory (Tianjin, China). Commercial 20 wt% Pt/C was bought from Johnson Matthey-J.M. (London, UK). All reagents and solvents are commercially available in this work and used without any further purification. Ultrapure water with a resistance of 18.2 MΩ cm (at 25 °C) was used for all experiments.

### 3.2. Synthesis of Electrocatalysts

Typically, hemin was dissolved in the 10 mM 50 mL NaOH solution with acid-treated commercial carbon (C-EC600) with a pH value of 10.1, and then hemin was reprecipitated and dispersed on C-EC600 by adding 3 M HCl solution. The final pH value of the solution is about 0.2, guaranteeing that most of the hemin was reprecipitated on the C-EC600. Here, we denoted the mixture as R-Hm/C-EC600. After that, 30 mg melamine and 150 mg cysteine were mixed with 175 mg ZnCl_2_ in the solution. The mixture was stirred at 80 °C until the solution was totally evaporated. At last, the mixture was further dried at 70 °C overnight. The obtained mixture was calcined in argon (Ar) at 500 °C for 2 h and a further 950 °C for 2 h. Finally, the resulting sample was marked as Fe-N,S-C-950 (Zn). In contrast, Fe-N,S-C-950 was fabricated under the same synthesis condition without adding ZnCl_2_. Fe-N-C-950 (Zn) was prepared using only melamine as the N-containing ligand.

### 3.3. Physical Characterizations

The physical characterization of catalysts was conducted with an X-ray diffractometer (X’pert Pro-1, PANalytical) from 10 to 90° (Almelo, The Netherlands), a Raman spectrometer (Bruker Optics Senterra, Ettlingen, Germany), a specific surface and pore size analysis instrument (QUADRASORB SI, Boynton Beach, FL, USA). The XPS of samples was conducted on Escalab Xi+ (Thermo Fisher Scientific, Waltham, MA, USA). TEM was carried out on TECNAI G2F30 (FEI Company, Hillsboro, OR, USA). The Fe content was measured by inductively coupled plasma optical emission spectroscopy (ICP-OES, ICPS-8100, Shimadzu, Japan).

### 3.4. Electrochemical Measurements

The as-synthetic NNMEs and the commercial Pt/C electrocatalyst were operated in a three-electrode system using CHI 760E (Chenhua, Shanghai, China) and VSP-300 (BioLogic, Paris, France) in 0.1 M KOH and 0.1 M HClO_4_ solutions. Here, glassy carbon electrodes (GCE, S = 0.19625 cm^2^) modified with electrocatalysts were used as working electrodes. A graphitic rod and a Hg/HgO or SCE were used as a counter and a reference electrode, respectively. All potentials were calibrated to the reversible hydrogen electrode (RHE) following the equation of E_RHE_ = E_Hg/HgO_ + 0.098 + 0.059pH in 0.1 M KOH and E_RHE_ = E_SCE_ + 0.244 + 0.059pH in 0.1 M HClO_4_ solutions. The resulting electrocatalysts or commercial Pt/C were dispersed in a mixed solution, containing ethanol, water and Nafion (5%) with a volume ratio of 9:1:0.06. After being sonicated for 10 mins, the electrocatalyst ink was repeatedly pipetted on the surface of GCE and dried in the air, subsequently obtaining the final electrocatalyst loading of 0.6 mg cm^−2^ in 0.1 M KOH and 1.0 mg cm^−2^ in 0.1 M HClO_4_ solutions for NNMEs, 20 µg_Pt_ cm^−2^ in 0.1 M KOH and 0.1 M HClO_4_ solutions for 20% Pt/C.

First, cyclic voltammetry (CV) curves were performed in the N_2_- and O_2_-saturated 0.1 M KOH and 0.1 M HClO_4_ solutions at a scanning rate of 100 mV s^−1^. Second, the rotating disk electrode (RDE) technique was conducted in the O_2_-saturated alkaline solution to collect the signal of linear sweep voltammograms (LSV) curves at a scanning rate of 5 mV s^−1^. Different rotation rates from 625 to 2025 rpm were collected at a scanning rate of 5 mV s^−1^ in alkaline and acidic solutions. After then, the Koutecky–Levich (K–L) equation was calculated for the electron transfer number (n) based on the LSV curves, as follows:

(1) $$\frac{1}{J}=\frac{1}{J_{K}}+\frac{1}{{B\omega }^{\frac{1}{2}}}$$

(2) $$B=0.62{\mathrm{nFD}}_{0}{}^{2/3}\nu^{-1/6}C_{0}$$

Here, J and J_k_ represent the measured and kinetic current density. F is the Faraday constant of 96,485 C mol^−1^. $C_{O_{2}}$ is the concentration of dissolved O_2_ as 1.22 × 10^−3^ and 1.26 × 10^−3^ mol cm^−3^ in 0.1 M KOH and 0.1 M HClO_4_, respectively [48]. $D_{O_{2}}$ of 1.93 × 10^−6^ cm^2^ s^−1^ represents the diffusion co-efficient of O_2_ in 0.1 M KOH and 0.1 M HClO_4_. ω and ν (0.01 cm^2^ s^−1^) are the rotation of RDE and kinematic viscosity of O_2_ in 0.1 M KOH and 0.1 M HClO_4_, respectively. A rotating ring-disk electrode (RRDE) was employed for detecting the electron transfer number (n) and %HO_2_^−^ in alkaline solutions or H_2_O_2_% in acidic solutions at a constant potential of 1.2 V (vs. RHE), based on the following equations:

(3) $${\mathrm{HO}}_{2}^{-}/H_{2}O_{2}\left(\%\right)=200\times \frac{\frac{I_{R}}{N}}{\left(\frac{I_{R}}{N}\right)+I_{D}}$$

(4) $$n=4\times \frac{I_{D}}{\left(\frac{I_{R}}{N}\right)+I_{D}}.$$

In the equations, I_R_ and I_D_ are the current of the ring and the current of the disk, respectively. N is the current collection efficiency of the Pt ring as 0.37 in this work.

All data were collected from the as-prepared cells at room temperature. In the synthesis of NNMEs-based cathodes, the electrocatalyst ink was fabricated by mixing 5 mg electrocatalysts with 10 mL 10% PTFE and 2 mL ethanol under ultra-sonification. The resulting slurry was further coated on the Ni foam to obtain a loading of 5 mg cm^−2^ and dried with an infrared lamp to remove residual solvent. A polished Zn plate was used as the anode and 6 M KOH solution was employed as the electrolyte. The assembled battery was conducted in air conditions.

## 4. Conclusions

In summary, an N and S co-doped electrocatalyst of Fe-N,S-C-950 (Zn) was synthesized by combining the self-assembly method with the evaporation strategy. After being modified with N and N,S-containing ligands with Zn, multiple Fe-based active sites were obtained after pyrolysis under a high temperature of 950 °C. Here, the Zn-induced electrocatalyst of Fe-N,S-C-950 (Zn) displays superior ORR performances in both 0.1 M KOH and 0.1 M HClO_4_ solutions. Meanwhile, as the air electrode in Zn-air batteries, Fe-N,S-C-950 (Zn) demonstrates a peak power density of 121.9 mW cm^−2^ with a high OCV (1.450 V). The supplement of S in Fe-N-C materials provides more FeS_x_ and the S-C species that can increase additional active sites for the enhancement of ORR performances. This work further improves the development of a Zn-assisted strategy in the assembly of porphyrins for the fabrication of NNMEs toward the ORR and ZABs.

## Data Availability

No data was shared.

## Supplementary Materials

The following supporting information can be downloaded at: https://www.mdpi.com/article/10.3390/molecules28155885/s1, Figure S1: the TEM images of Fe-N,S-C-950 (Zn); Figure S2: the HR-TEM images of Fe-N,S-C-950 (Zn); Figure S3: the high-resolution XP spectra of N 1s of (a) Fe-N-C-950 (Zn) and (b) Fe-N,S-C-950; Figure S4: the N content relative to C of Fe-N,S-C-950, Fe-N,S-C950 (Zn) and Fe-N-C-950 (Zn); Figure S5: the CV curves of Fe-N,S-C-950 (Zn) in the N_2_ and O_2_-saturated 0.1 M KOH solutions; Table S1: the specific surface area of Fe-N,S-C-950, Fe-N,S-C-950 (Zn) and Fe-N-C950 (Zn); Table S2: the fitting parameters of high-resolution XP spectra of N1s for Fe-N,S-C-950, Fe-N,S-C-950 (Zn) and Fe-N-C-950 (Zn); Table S3: the ORR performances in comparison with Fe-N,S-C-950 (Zn) and other representative NNMEs for ORR in alkaline solutions; Table S4: the comparison of Fe-N,S-C-950 (Zn) with advanced NNMEs for ZAB performances. References [49,50,51,52,53,54,55,56,57,58,59,60,61,62,63,64,65,66,67,68,69,70,71,72] are cited in the supplementary materials.
