# Supplementary material for: Zn-Induced Synthesis of Porous Fe-N,S-C Electrocatalyst with Iron-Based Active Sites Containing Sulfides, Oxides and Nitrides for Efficient Oxygen Reduction and Zinc-Air Batteries
Source: Molecules. 2023 Aug 4;28(15):5885. doi: 10.3390/molecules28155885 (PMC10421323; doi:10.3390/molecules28155885)
Supplement: Supplementary file 1 [file molecules-28-05885-s001.zip › molecules-2487970-supplementary.pdf]

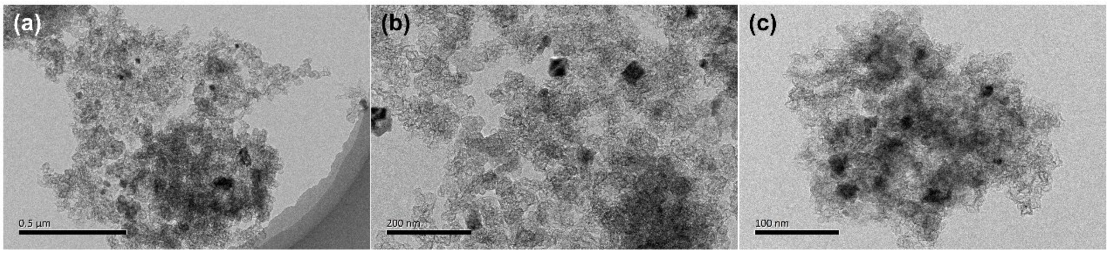

Zn-Induced Synthesis of Porous Fe-N,S-C Electrocatalyst with Iron Based Active Sites

Figure S1. The TEM images of Fe-N,S-C-950 (Zn).

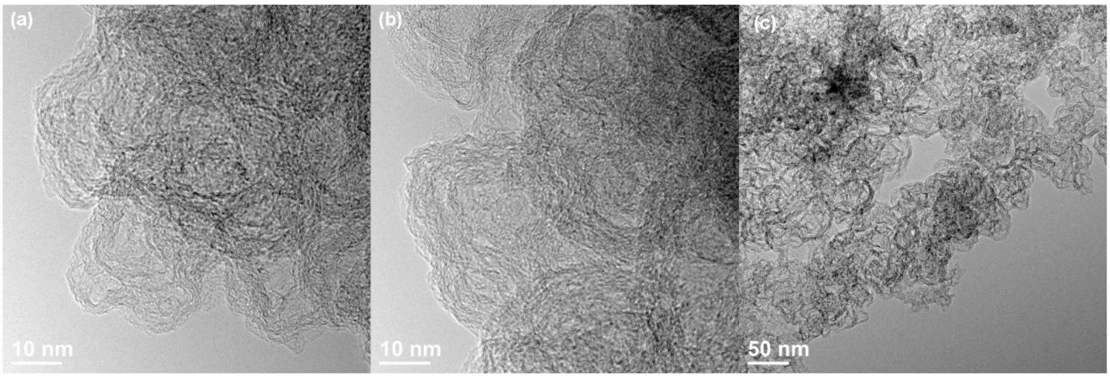

Figure S2. The HR-TEM images of Fe-N,S-C-950 (Zn).

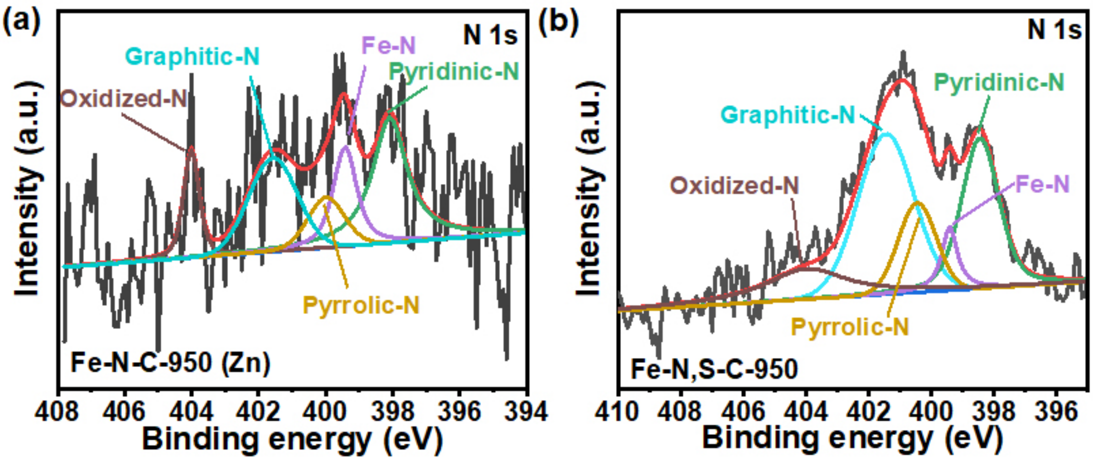

Figure S3. The high-resolution N 1s of (a) Fe-N-C-950 (Zn) and (b) Fe-N,S-C-950.

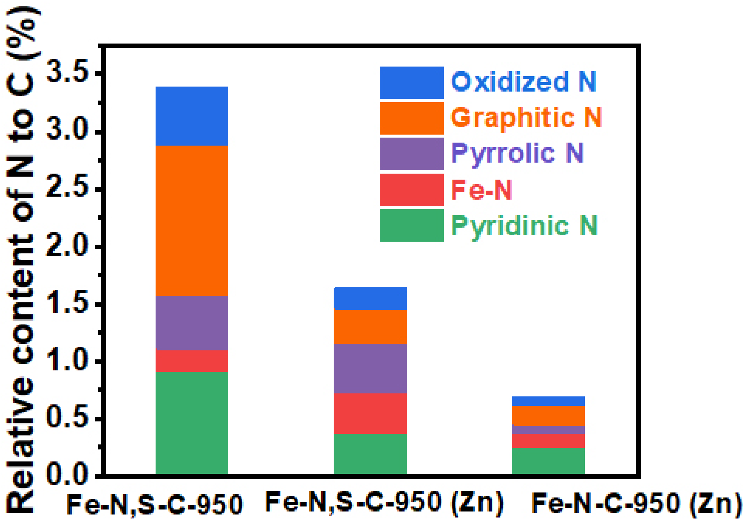

Figure S4. The N content relative to C of electrocatalysts Fe-N,S-C-950, Fe-N,S-C-950 (Zn) and Fe-N-C-950 (Zn).

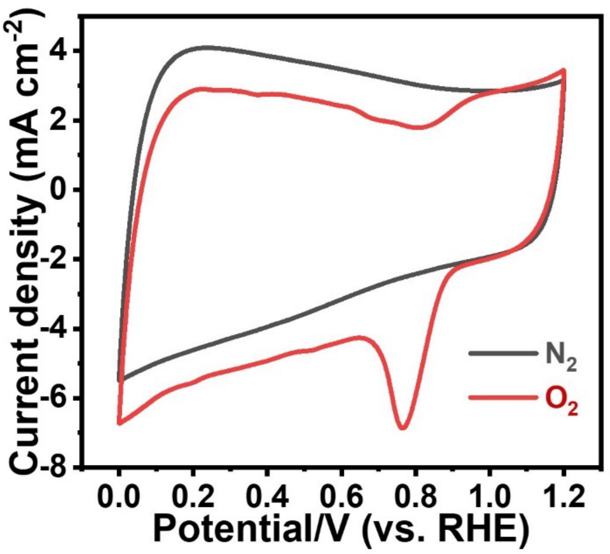

Figure S5. The CV curves of Fe-N,S-C-950 (Zn) in N_2 and O_2-saturated 0.1 M KOH solution.

Table S1. The specific surface area of Fe-N,S-C-950, Fe-N,S-C-950 (Zn) and Fe-N-C-950 (Zn).

Sample	BET Surface Area (m^2 g^{-1})	t-Plot Micropore Area (m^2 g^{-1})	t-Plot External Surface Area (m^2 g^{-1})
**Fe-N,S-C-950**	669.0	168.5	500.5
**Fe-N,S-C-950 (Zn)**	951.5	172.8	778.7
**Fe-N-C-950 (Zn)**	801.2	64.2	737.0

Table S2. The fitting parameters of high-resolution XPS N1s spectra of Fe-N,S-C-950, Fe-N,S-C-950 (Zn) and Fe-N-C-950 (Zn).

Catalysts	Binding energy of N species (eV) and content (%)					N relative to C (at%)
	Pyridinic N	Fe-N	Pyrrolic N	Graphitic N	Oxidized N	
**Fe-N,S-C-950**	398.5 (27.2)	399.4 (6.1)	400.5 (14.0)	401.5 (37.6)	404.0 (15.1)	3.39
**Fe-N,S-C-950 (Zn)**	398.3 (23.0)	399.2 (21.3)	400.7 (26.4)	401.6 (18.4)	404.0 (10.9)	1.64
**Fe-N-C-950 (Zn)**	398.1 (35.6)	399.4 (17.1)	400.0 (10.9)	401.6 (24.9)	404.0 (11.5)	0.69

Table S3. The ORR performances in comparison with Fe-N,S-C-950 (Zn) and other representative NNMEs for ORR in alkaline solution.

Electrocatalyst	Onset potential (V vs. RHE)	Half-wave potential ($E_{1/2}$) (V vs. RHE)	Limiting current density (J_L mA cm^{-2})	Reference
CoZn-NC-700	0.98	0.84	4.93	[1]
OM-NCNF-FeN_x	0.905	0.836	5.6	[2]
Fe/Mn-N-C	1.02	0.880	5.7	[3]

Fe_1-HNC-500-850	0.93	0.842	5.80	[4]
Fe_3C@NCNTs	0.92	0.840	5.80	[5]
ZFP-800	1.028	0.875	5.86	[6]
CoZn/CNT@Por.NC	-	0.87	5.55	[7]
FeCoP@NMn-CNS-800	1.02	0.84	5.81	[8]
ZnS/NSC-NaCl	1.02	0.905	6.32	[9]
CoN/CNCs-800	0.964	0.852	5.18	[10]
750-SAHm/EC600	1.0	0.89	~6.5	[11]
Fe/NSC (800)	0.84	0.76	~5	[12]
FeNSC-850	0.89	0.82	~5.2	[13]
**Fe-N,S-C-950 (Zn)**	**0.921**	**0.844**	**6.03**	**This work**

Table S4. Performance comparison of Fe-N,S-C-950 (Zn) with advanced NNMEs for ZABs.

Electrocatalyst	Open-circuit voltage (V)	Maximum power density (mW cm^{-2})	Specific capacity (mAh g^{-1})	Reference
**Fe-N,S-C-950 (Zn)**	**1.450**	**121.9**	**639**	**This work**
Fe/Fe_3C@FeNC	1.414	134.6	856.2	[14]
Fe_3O_4@NHCS-2	1.42	133	701	[15]
Fe_2O_3@NC-450	1.267	156.6	-	[16]
Fe_3C/Fe_2O_3@NGNs	1.46	139.8	722	[17]
FePPc@CB	1.367	90	1391	[18]
FeS/Fe_3C@NS-C-900	1.455	192.2	750	[19]
Fe-N-C	1.48	175	775.7	[20]
FeSA-FeNC@NSC	1.48	259.88	811.03	[21]
ISG Fe-N-C	1.48	259.1	763	[22]
Fe-SAs/Fe_3C-Fe	1.42	158	762	[23]
FePc&rGO	1.48	103	739.7	[24]
CoFe@NCS-24	1.477	146	812	[25]
320-$Fe_1Co_{1.5}$-NC-800	1.499	77	-	[26]
NP-CoSANC	1.42	158.1	768.4	[27]
SAs-Fe/N-CNS	1.45	157.03	789.82	[28]
CoN-Nd	1.44	120	702	[29]
SPE-FeN_x-HPNC	1.5	150	748	[30]

Reference

1. Chen, B.; He, X.; Yin, F.; Wang, H.; Liu, D.J.; Shi, R.; Chen, J., Yin, H., MO-Co@ N-doped carbon (M= Zn or Co): vital roles of inactive Zn and highly efficient activity toward oxygen reduction/evolution reactions for rechargeable Zn−air battery, *Adv. Funct. Mater.,* **2017**, 27, 1700795.

2. Cheng, C.; Li, S.; Xia, Y.; Ma, L.; Nie, C.; Roth, C.; Thomas, A., Haag, R.J.A.m., Atomic Fe–N_x coupled open-mesoporous carbon nanofibers for efficient and bioadaptable oxygen electrode in Mg–air

batteries, *Adv. Mater.,* **2018**, 30, 1802669.
3. Chen, Z.; Liao, X.; Sun, C.; Zhao, K.; Ye, D.; Li, J.; Wu, G.; Fang, J.; Zhao, H., Zhang, J., Enhanced performance of atomically dispersed dual-site Fe-Mn electrocatalysts through cascade reaction mechanism, *Appl. Catal. B-Environ.,* **2021**, 288, 120021.
4. Zhang, X.; Zhang, S.; Yang, Y.; Wang, L.; Mu, Z.; Zhu, H.; Zhu, X.; Xing, H.; Xia, H., Huang, B., A general method for transition metal single atoms anchored on honeycomb-like nitrogen-doped carbon nanosheets, *Adv. Mater.,* **2020**, 32, 1906905.
5. Xu, C.; Guo, C.; Liu, J.; Hu, B.; Dai, J.; Wang, M.; Jin, R.; Luo, Z.; Li, H., Chen, C., Accelerating the oxygen adsorption kinetics to regulate the oxygen reduction catalysis via Fe_3C nanoparticles coupled with single $Fe-N_4$ sites, *Energy Storage Mater.,* **2022**, 51, 149-158.
6. Zhang, T.; Mao, S.; Sun, P.; Gao, X.; Fang, H.; Luo, H.; Zhang, W., Zhou, B., Nanosized FeS/ZnS heterojunctions derived using zeolitic imidazolate Framework-8 (ZIF-8) for pH-universal oxygen reduction and High-efficiency Zn–air battery, *J. Colloid Interf. Sci.,* **2022**, 608, 446-458.
7. Liang, J.; Chen, J.; Wang, G.; Liu, J.; Wang, N.; Shi, Z., Interfaces, Hydrogel-derived Co_3ZnC/Co nanoparticles with heterojunctions supported on N-doped porous carbon and carbon nanotubes for the highly efficient oxygen reduction reaction in Zn–air batteries, *ACS Appl. Mater. Interfaces,* **2022**, 14, 48789-48800.
8. Chen, Y.-P.; Lin, S.-Y.; Sun, R.-M.; Wang, A.-J.; Zhang, L.; Ma, X., Feng, J.-J., FeCo/FeCoP encapsulated in N, Mn-codoped three-dimensional fluffy porous carbon nanostructures as highly efficient bifunctional electrocatalyst with multi-components synergistic catalysis for ultra-stable rechargeable Zn-air batteries, *J. Colloid Interf. Sci.,* **2022**, 605, 451-462.
9. Cui, L.; Xiang, K.; Kang, X.; Zhi, K.; Wang, L.; Zhang, J.; Fu, X.-Z., Luo, J.-L., ZnS anchored on porous N, S-codoped carbon as superior oxygen reduction reaction electrocatalysts for Al-air batteries, *J. Colloid Interf. Sci.,* **2022**, 609, 868-877.
10. Zhang, X.; Huang, W.; Zhang, J.; Wang, Y.; Astruc, D., Liu, X., Facile synthesis of three-dimensional Co/N co-doped carbon nanocuboids for an enhanced oxygen reduction reaction, *Inorg. Chem. Front.,* **2023**, 10, 1739-1747.
11. Shen, S.; Zhai, Z.; Qin, J.; Zhang, X., Song, Y., Pyrolysis of self-assembled hemin on carbon for efficient oxygen reduction reaction, *J. Porphyr. Phthalocyanines*
2019, 23, 1013-1019.
12. Zhao, H.; Chen, L.; Xu, Y.; Wang, H.; Li, J.-Y.; Xie, Y., Wang, L., A nitrogen and sulfur co-doped iron-based electrocatalyst derived from iron and biomass ligand towards the oxygen reduction reaction in alkaline media, *Dalton Trans.,* **2021**, 50, 13943-13950.
13. Liu, Y.; Xu, Y.; Wang, H.; Zhang, J.; Zhao, H.; Chen, L.; Xu, L.; Xie, Y., Huang, J., MIL-88-derived N and S co-doped carbon materials with supplemental FeS_x to enhance the oxygen reduction reaction performance, *Catalysts,* **2022**, 12, 806.
14. Huang, N.; Dong, W.; Feng, Y.; Liu, W.; Guo, L.; Xu, J., Sun, X., Using dopamine interlayers to construct Fe/Fe_3C@FeNC microspheres of high N-content for bifunctional oxygen electrocatalysts of Zn-air batteries, *Dalton Trans.,* **2023**, 52, 2373-2383.
15. Li, Y.; Huang, H.; Chen, S.; Yu, X.; Wang, C., Ma, T., 2D nanoplate assembled nitrogen doped hollow carbon sphere decorated with Fe_3O_4 as an efficient electrocatalyst for oxygen reduction reaction and Zn-air batteries, *Nano Res.,* **2019**, 12, 2774-2780.
16. Xiao, Z.; Wu, C.; Wang, W.; Pan, L.; Zou, J.; Wang, L.; Zhang, X., Li, G., Tailoring the hetero-structure of iron oxides in the framework of nitrogen doped carbon for the oxygen reduction

reaction and zinc-air batteries, *J. Mater. Chem. A,* **2020**, 8, 25791-25804.
17. Tian, Y.; Xu, L.; Qian, J.; Bao, J.; Yan, C.; Li, H.; Li, H., Zhang, S., Fe_3C/Fe_2O_3 heterostructure embedded in N-doped graphene as a bifunctional catalyst for quasi-solid-state zinc-air batteries, *Carbon,* **2019**, 146, 763-771.
18. Cheng, W.-Z.; Liang, J.-L.; Yin, H.-B.; Wang, Y.-J.; Yan, W.-F., Zhang, J.-N., Bifunctional iron-phtalocyanine metal-organic framework catalyst for ORR, OER and rechargeable zinc-air battery, *Rare Metals,* **2020**, 39, 815-823.
19. Li, Y.-W.; Zhang, W.-J.; Li, J.; Ma, H.-Y.; Du, H.-M.; Li, D.-C.; Wang, S.-N.; Zhao, J.-S.; Dou, J.-M., Xu, L., Fe-MOF-derived efficient ORR/OER bifunctional electrocatalyst for rechargeable zinc-air batteries, *ACS Appl. Mater. Interfaces,* **2020**, 12, 44710-44719.
20. Lu, X.; Xu, H.; Yang, P.; Xiao, L.; Li, Y.; Ma, J.; Li, R.; Liu, L.; Liu, A.; Kondratiev, V.; Levin, O.; Zhang, J., An, M., Zinc-assisted MgO template synthesis of porous carbon-supported Fe-Nx sites for efficient oxygen reduction reaction catalysis in Zn-air batteries, *Appl. Catal. B-Environ.,* **2022**, 313, 121454.
21. Zhai, W.; Huang, S.; Lu, C.; Tang, X.; Li, L.; Huang, B.; Hu, T.; Yuan, K.; Zhuang, X., Chen, Y., Simultaneously integrate iron single atom and nanocluster triggered tandem effect for boosting oxygen electroreduction, *Small,* **2022**, 18, 2107225.
22. Liu, M.; Wang, L.; Zhang, L.; Zhao, Y.; Chen, K.; Li, Y.; Yang, X.; Zhao, L.; Sun, S., Zhang, J., In-situ silica xerogel assisted facile synthesis of Fe-N-C catalysts with dense Fe-Nx active sites for efficient oxygen reduction, *Small,* **2022**, 18, 2104934.
23. Sun, X.; Wei, P.; Gu, S.; Zhang, J.; Jiang, Z.; Wan, J.; Chen, Z.; Huang, L.; Xu, Y.; Fang, C.; Li, Q.; Han, J., Huang, Y., Atomic-level Fe-N-C coupled with Fe_3C-Fe nanocomposites in carbon matrixes as high-efficiency bifunctional oxygen catalysts, *Small,* **2020**, 16, 1906057.
24. Mei, Z.-y.; Cai, S.; Zhao, G.; Jing, Q.; Sheng, X.; Jiang, J., Guo, H., Understanding electronic configurarions and coordination environment for enhanced ORR process and improved Zn-air battery performance, *Energy Stor. Mater.,* **2022**, 50, 12-20.
25. Cheng, H.; Zhuang, Y.; Meng, C.; Chen, B.; Chen, J.; Yuan, A., Zhou, H., Ultrafine CoFe nanoparticles supported on nitrogen-doped carbon sheets boost oxygen electrocatalysis for Zn-air batteries, *Appl. Surf. Sci.,* **2023**, 607, 154953.
26. Zhang, Y.; Zhao, M.; Yang, Q.; Lai, M.; Zhang, J.; Liu, C.; Xu, X., Jia, J., Agarose-gel-based self-limiting synthesis of a bimetal (Fe and Co)-doped composite as a bifunctional catalyst for a zinc-air battery, *J. Colloid Interf. Sci.,* **2023**, 635, 186-196.
27. Rong, J.; Gao, E.; Liu, N.; Chen, W.; Rong, X.; Zhang, Y.; Zheng, X.; Ao, H.; Xue, S., Huang, B., Porphyrinic MOF-derived rich N-doped porous carbon with highly active CoN_4C single-atom sites for enhanced oxygen reduction reaction and Zn-air batteries performance, *Energy Stor. Mater.,* **2023**, 56, 165-173.
28. Gong, X.-F.; Zhang, Y.-L.; Zhao, L.; Dai, Y.-K.; Cai, J.-J.; Liu, B.; Guo, P.; Zhou, Q.-Y.; Yagi, I., Wang, Z.-B., Zinc/graphitic carbon nitride co-mediated dual-template synthesis of densely populated Fe-Nx-embedded 2D carbon nanosheets towards oxygen reduction reactions for Zn-air batteries, *J. Mater. Chem. A,* **2022**, 10, 5971-5980.
29. Hu, Y.; Guo, M.; Hu, C.; Dong, J.; Yan, P.; Isimjan, T.T., Yang, X., Engineering cobalt nitride nanosheet arrays with rich nitrogen defects as a bifunctional robust oxygen electrocatalyst in rechargeable Zn-air batteries, *J. Colloid Interf. Sci.,* **2022**, 608, 2066-2074.
30. Lu, X.; Xiao, L.; Yang, P.; Xu, H.; Liu, L.; Li, R.; Li, Y.; Zhang, H.; Zhang, J., An, M., Highly exposed

surface pore-edge FeNx sites for enhanced oxygen reduction performance in Zn-air batteries, *Inorg. Chem. Front.*, **2023**, 10, 815–823.
